# Dielectric Metalens: Properties and Three-Dimensional Imaging Applications

**DOI:** 10.3390/s21134584

**Published:** 2021-07-04

**Authors:** Sun-Je Kim, Changhyun Kim, Youngjin Kim, Jinsoo Jeong, Seokho Choi, Woojun Han, Jaisoon Kim, Byoungho Lee

**Affiliations:** 1 Department of Physics, Myongji University, Myongjiro 116, Namdong, Cheoin-gu, Yongin 17058, Korea; choisegho@naver.com (S.C.); maxwell59@naver.com (W.H.); 1010jskim@gmail.com (J.K.); 2 Inter-University Semiconductor Research Center, School of Electrical and Computer Engineering, Seoul National University, Gwanak-Gu Gwanakro 1, Seoul 08826, Korea; kch3782@snu.ac.kr (C.K.); ttw8592@snu.ac.kr (Y.K.); byoungho@snu.ac.kr (B.L.); 3 Hologram Research Center, Korea Electronics Technology Institute, 8 Floor, 11, World cup buk-ro 54-gil, Mapo-gu, Seoul 13488, Korea; j.jeong@keti.re.kr

**Keywords:** metasurface, dielectric metalens, aberrations, flat optics, three-dimensional imaging, display, sensing, augmented and virtual realities, depth sensing, light analysis

## Abstract

Recently, optical dielectric metasurfaces, ultrathin optical skins with densely arranged dielectric nanoantennas, have arisen as next-generation technologies with merits for miniaturization and functional improvement of conventional optical components. In particular, dielectric metalenses capable of optical focusing and imaging have attracted enormous attention from academic and industrial communities of optics. They can offer cutting-edge lensing functions owing to arbitrary wavefront encoding, polarization tunability, high efficiency, large diffraction angle, strong dispersion, and novel ultracompact integration methods. Based on the properties, dielectric metalenses have been applied to numerous three-dimensional imaging applications including wearable augmented or virtual reality displays with depth information, and optical sensing of three-dimensional position of object and various light properties. In this paper, we introduce the properties of optical dielectric metalenses, and review the working principles and recent advances in three-dimensional imaging applications based on them. The authors envision that the dielectric metalens and metasurface technologies could make breakthroughs for a wide range of compact optical systems for three-dimensional display and sensing.

## 1. Introduction

Over the last decade, optical dielectric metasurfaces, ultrathin optical skins with densely arranged dielectric nano-antennas, have been in the spotlight in the optics and photonics communities from both industry and academia [1,2,3]. The reason for such explosive interest is based on the unprecedented, revolutionary properties of dielectric metasurfaces such as ultrathin thickness and light weight, large degree of freedom in optical wavefront encoding, suppression of higher order diffraction, polarization tunability, large diffraction angle, and ease of fabrication and integration. By virtue of the advantages, it has turned out that dielectric metasurfaces-based flat optic elements prevail in many aspects of light manipulation performances, compared to conventional refractive and diffractive optic elements [4,5].

While flat metasurface optics technology has been going through rapid progress, there have been many academic efforts to exploit dielectric metasurfaces for next-generation imaging applications requiring small form factor, multi-functionality, wide spatial frequency modulation bandwidth, and large design degree of freedom. Thus, nowadays, many researchers are expecting that dielectric meta-optics technologies could revolutionize the three-dimensional imaging applications for display and optical sensing, including wearable and mobile electronic devices, near-eye displays, security, and remote sensing for automotive car and drones [6,7]. In particular, among many flat meta-optic elements [1,2,3] including the spectral filter, polarizer, waveplate, angular filter, and beam deflection prism, the dielectric metasurface lens also called dielectric metalens is regarded as the core of flat optics owing to its advanced compact imaging capability [8,9,10].

In this paper, the basic properties and performance of dielectric metalenses are introduced briefly. In addition, the recent efforts for their use in the three-dimensional imaging applications of display and sensing technologies, is reviewed. At first, basic working principles and design methods of dielectric metalens are described in the Section 2. Second, in Section 3, imaging performances of metalenses are discussed by introducing numerous representative studies. Then, the recent advances in metalens imaging applications would be introduced with a few selected publications, focusing on advanced three-dimensional display and depth sensing technologies. At last, discussion and perspectives on the future of dielectric metalens technology would be suggested as concluding remarks. Even though there are several review papers dealing with the principles of metasurfaces and their applications [1,2,3,4,5,6], a thorough review article focusing on optical imaging performances of dielectric metalens and their use in three-dimensional imaging applications are missing to the best of our knowledge. Thus, our main focus is to introduce imaging properties of metalenses and to reveal their roles and future directions in on-demand three-dimensional imaging functions.

## 2. Basic Principles of Dielectric Metalenses

A dielectric metalens consists of a quasi-periodic array of subwavelength sized dielectric nanoantennas with spatially varying geometries placed on a transparent substrate. Dielectric nanoantennas can modulate scattered (transmitted or reflected) local phase according to the transverse positions of nanoantennas, almost independently from neighboring ones. In general, nanoantennas are structured as high contrast posts made of dielectrics (insulators or semiconductors) with large refractive index and low extinction coefficient in a target spectral range. For visible light, TiO_2_, GaN, c-Si, SiN, and a-Si:H are widely used material candidates while a-Si is the best candidate in near-infrared range. Once a physical encoding method is chosen to design geometry of each nanoantenna, optical phase can be freely encoded for a certain wavelength without higher order diffractions. The basic hyperbolic phase profile for on-axis spherical lensing can be modelled regarding optical phase difference as follows [9].

(1) $$\varphi (x,y)=\frac{2\pi }{\lambda_{d}}(f-\sqrt{x^{2}+y^{2}+f^{2}}).$$

In Equation (1), *λ_d_*, *f*, *x*, and *y* refer to the design wavelength, focal length, and sampled in-plane position coordinates of a metalens. Basically, dielectric nanoantennas are assumed to act as truncated dielectric monomode waveguides of the same height, with or without an anisotropic property. The period (unitcell spacing) of nanoantenna array should be smaller than Nyquist sampling limit (*λ*/2NA) to suppress higher order diffraction loss, where *λ* and NA are operation wavelength and numerical aperture, respectively.

There are two different general methods for a local nanoantenna (unitcell) geometry design to achieve required phase modulation at a certain sampling position. One is to design local rotation direction of anisotropic nanoantenna based on geometric phase, also called Pancharatnam–Berry phase [8,9]. Another is to tune propagation phase delay by controlling cross-section dimension of isotropic nanonatenna waveguides and follow their effective mode indices [10].

**Figure 1:**
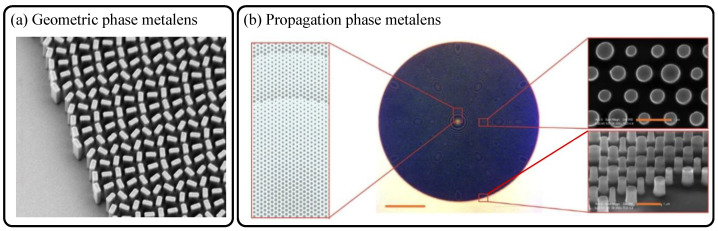
(**a**) Scanning electron microscope image of the geometric-phase-based TiO_2_ nanofin metalens reported in 2016 [9]. (**b**) Optical micrograph (scale bar: 100 μm) and scanning electron microscope images of the propagation-phase-based a-Si nanopost metalens reported in 2015 [10]. The scalebars in inset electron beam images of (**b**) refer to 1 μm. (**a**) Reprinted with permission from [9]. Copyright 2016 The American Association for the Advancement of Science. (**b**) Reprinted with permission from [10]. Copyright 2015 Springer Nature.

In 2015, dielectric metasurfaces and metalens were reported by A. Arbabi et al. using elliptical amorphous silicon nanoposts for vectorial wavefront control and focusing of near-infrared light for the first time, to the best of our knowledge [8]. In 2016, as depicted in Figure 1a, dielectric metalens made of TiO_2_ nanofins was proposed for focusing and diffraction-limited imaging of visible light by M. Khorasaninejad et al. [9]. These two studies applied geometric phase method in common. The principle of geometric phase is to tune phase of cross-polarized transmission rather than co-polarized one under a circularly polarized illumination. Equation (2) below shows analytical description of transmitted electric field vector tuned by rotation angle, *θ*(*x,y*), dependent geometric phase. Here, each nanoantenna (nanofin or nanorod) acts as a nanoscale local waveplate with spatially variant principal axes.

(2) $${\vec{E}}_{t}=\eta_{co}\left(\begin{array}{l} 1\\ \pm i \end{array}\right)+\eta_{cross}e^{2j\theta (x,y)}\left(\begin{array}{l} 1\\ \mp i \end{array}\right)$$

*η*_co_ and *η*_cross_ denote complex amplitudes of co- and cross-polarized transmissions. It is worth noting that the geometric phase does not depend on dimension of nanoantenna, material property, and operation wavelength. The modulation of cross-polarized light is only achieved via 2*θ*(*x,y*) term from degree of local nanoantenna rotation for encoding *φ*(*x*,*y*), and full 2π phase can be easily covered by rotating nanofin or nanorod with angle of π.

On the other hand, the method of propagation phase is simply tuned by changing dimension of nanoantenna cross-section. In this case, phase modulation without polarization modulation is described as ${\vec{E}}_{t}=n_{eff}(x,y,\lambda )H{\vec{E}}_{in}$, where λ, *H*, and *n_eff_* refer to operation wavelength, height of nanoantenna and effective index of the fundamental propagation mode, respectively. In this case, local abrupt phase delay is given by *φ*(*x*,*y*) = 2π*n_eff_H*/*λ*, and full 2π phase modulation can be achieved using the condition of Equation (3) below.

(3) $$H=\frac{\lambda }{n_{eff,m}-1}.$$

Here, *n_eff,m_* is the maximum effective fundamental mode index for the nanopost with the highest fill factor in a single pixel. By contrast with the geometric phase, the propagation control method exhibits wavelength dependency. In the work by A. Arbabi et al. [10] reported in 2015, the isotropic silicon nanopost-based hyperbolic metalens described in Figure 1b has been demonstrated based on propagation phase modulation.

In addition, there are many different, multi-functional wavefront generation methods of hybridizing different spatial phase profiles and multiplexing multiple nanoantennas in a single device with a shared aperture. For example, multi-foci metalenses can be efficiently demonstrated with a large degree of freedom in designating positions and polarizations of foci [11,12,13,14].

## 3. Focusing and Imaging Performance of Dielectric Metalenses

### 3.1. Performances: Focusing and Imaging Qualities

**Figure 2:**
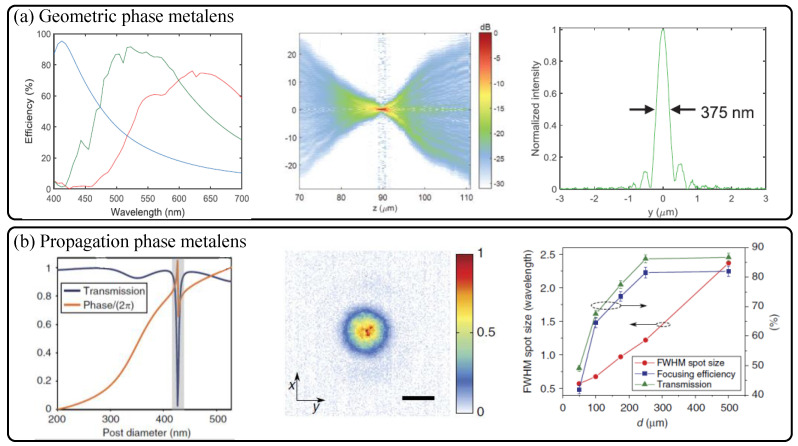
Similar high numerical aperture (NA) diffraction-limited on-axis focusing performances of (**a**) the geometric phase based nanofin metalens by F. Capasso group [9] and (**b**) the propagation phase based isotropic nanopost metalens by the A. Faraon group [10]. The left sub-figure in (**a**) shows cross-polarization efficiency of the three types of TiO_2_ nanofin pixels. The central sub-figure of (**a**) shows measured focusing map in image space, and the right sub-figure refers to the cross-sectional point spread function at the focal point (NA: 0.8) while the collimated incident light with the wavelength of 532 nm is illuminated. In (**b**), transmission amplitude and phase response of the a-Si nanopost (**left**) according to diameter variation, point spread function at the focal point (**center**), and image position (*d*) dependent plots of focal spot size and efficiency (**right**), at the wavelength of 1550 nm, respectively. In the rightmost figure in (**b**), increase of designed *d* implies decrease of NA and increase of focal length. The scale bar of the central point spread function in (**b**) denotes 1 μm. (**a**) Reprinted with permission from [9]. Copyright 2016 The American Association for the Advancement of Science. (**b**) Reprinted with permission from [10]. Copyright 2015 Springer Nature.

Upon understanding about the working principles discussed above, discussion of focusing and imaging performances is the essential next step. Here, we first focus on the performance of a planar dielectric metalens singlet with basic hyperbolic and other optimized phase profiles. Figure 2 shows the on-axis monochromatic focusing properties of the two different representative early metalenses designed for spherical wavefront generation [9,10]. Throughout the paper, we call the metalenses forming spherical wavefronts as the hyperbolic metalenses reflecting their phase profiles. Owing to the broadband characteristics of dielectric nanoantennas for the geometric phase and the propagation phase shown in the leftmost figures in Figure 2a,b, the two different hyperbolic metalenses show nearly diffraction-limited focusing with large efficiency and small focal spot size reaching theoretical limit of *λ*/2NA. The NA of the metalenses is calculated via conventional definition, $NA=\left(D/2\right)/\sqrt{f^{2}+{\left(D/2\right)}^{2}}\approx D/2f$. The approximation is valid for low NA values considering paraxial rays. The on-axis high NA (0.8) focusing efficiencies of the TiO_2_ metalens reach 70% at the target green wavelength (532 nm) in the visible [9], and similar highly efficient and NA dependent focusing efficiency is achieved with a-Si nanopost metalens as shown in the right figure in Figure 2b [10].

**Figure 3:**
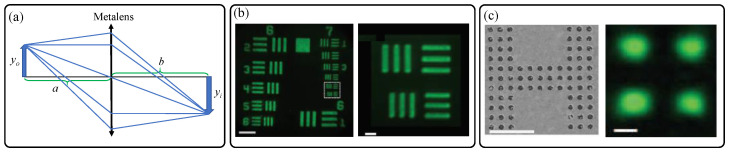
(**a**) Schematic diagram describing the simple object imaging relation of a flat hyperbolic metalens. (**b**,**c**) Nearly diffraction-limited object imaging performances of the TiO_2_ nanofin metalens by Capasso group in 2016 [9]. Experimentally captured camera images of (**b**) 1951 United States Air Force (USAF) resolution test chart and (**c**) nano-scale metallic hole arrays, formed by the TiO_2_ nanofin metalens at the wavelength of 532 nm. The scale bars in (**b**) and (**c**) denote 40 μm, 5 μm, 10 μm, and 500 nm, from the left to right, respectively. (**b**,**c**) Reprinted with permission from [9]. Copyright 2016 The American Association for the Advancement of Science.

Figure 3a graphically describes conventional thin lens imaging relations of 1/a+1/b=1/f, where *a*, *b*, and *f* denote positions of object and image, and focal length, respectively. This simple paraxial imaging relation is still valid for a hyperbolic metalens, and more appropriate for tracing rays with high incidence angle, since a hyperbolic metalens is ideally free from spherical aberration at the design wavelength (See Equation (1)). Based on such imaging properties and the aforementioned excellent high NA focusing performances, microscopic samples are successfully imaged using the TiO_2_ metalens as shown in Figure 3b,c). In particular, Figure 3c shows that the singlet hyperbolic metalens can resolve two nanoholes (subwavelength separation distance: 450 nm) close to the Abbe’s diffraction limit.

### 3.2. Aberrations of Dielectric Metalenses

In this section, we focus on the aberration characteristics of dielectric metalenses and correction methods of such aberrations, by introducing the recent studies on these topics. Conventionally, the major aberrations of a lens can be typically classified into two categories of chromatic and monochromatic aberrations according to their chromaticity. When it comes to monochromatic aberrations, there are the five Seidel’s aberrations which were denominated in a view of geometric optics, spherical aberration, coma, astigmatism, field curvature, and distortion [15].

**Figure 4:**
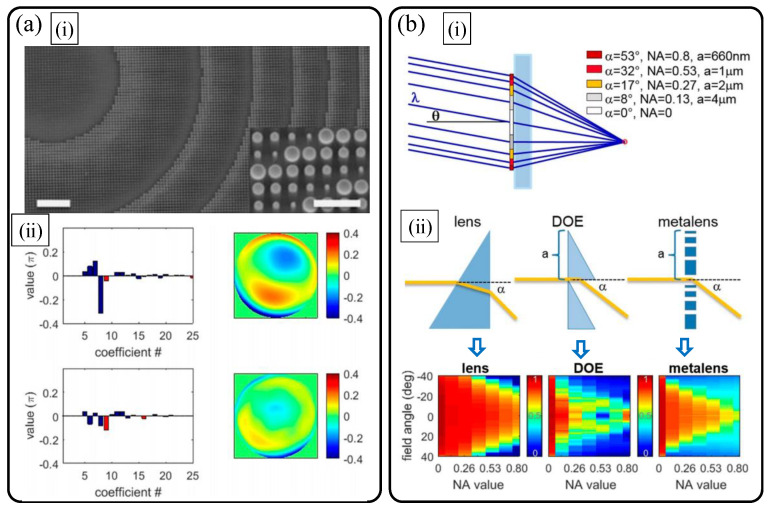
Performance limits of a flat hyperbolic TiO_2_ dielectric metalens [16]. (**a**) (**i**) Scanning electron micrographs and (**ii**) on-axis wavefront aberration comparison of polarization insensitive hyperbolic metalens (lower plots) with reference objective lens (upper plots) with the same NA of 0.8. The two scale bars in (**i**) are 5 μm (**left**) and 1 μm (**right**), respectively. In (**ii**), left and right plots are Zernike aberration coefficients with the Fringe notation (red: spherical aberrations) and normalized wavefront error function maps, respectively. (**b**) Field and wavelength-dependent focusing efficiency: comparison of the hyperbolic metalens with refractive and diffractive lenses. (**i**) Simulation scheme and (**ii**) simulated deflection efficiencies of refractive, diffractive, and metasurface lenses according to field angle. (**a**,**b**) Reprinted with permission from [16]. Copyright 2019 American Chemical Society.

First, it is important to discuss the aberrations and follow the performance limits of a hyperbolic dielectric metalens. In 2019, M. Decker et al. from Carl Zeiss and Capasso group of Harvard University reported a rigorous systematic study on imaging performance of a hyperbolic TiO_2_ nanopost metalens following a hyperbolic phase profile in Equation (1) [16]. In this paper, the main concern is focused on the investigation of general properties of spectral and angular bandwidth of a hyperbolic dielectric metalens using wavefront aberration function based on Zernike function decomposition [17,18] rather than simple comparison of NA and the Strehl ratio. When it comes to the on-axis imaging performance, at the design wavelength, the millimeter scale high-NA (0.8) TiO_2_ metalens shown in (i) in Figure 4a outperforms the reference objective with the same NA, and shows negligible coma and a little spherical aberration in the measurement and phase retrieval results as seen in (ii) in Figure 4a. If the operation wavelength and field angle deviate from the design wavelength and optic axis, interesting performance degradation occurs.

In the case of chromatic aberration induced by wavelength deviation, significant spherical aberration occurs and NA decreases largely (in the paper, from 0.8 to 0.5). It is the inherent characteristic of dielectric metalens that originates from strong light-matter interaction inside dielectric nanoantenna waveguides and enhanced material dispersion of high index materials inside them.

When considering oblique fields, among many monochromatic aberrations, the coma shows a distinguished contribution for the design wavelength in the TiO_2_ metalens. In contrast, for deviated colors, spherical aberration becomes dominant while the coma is suppressed. At last, these combinatoric spectral and angular aberration characteristics are rigorously compared with conventional refractive and diffractive lenses via simulations (Figure 4b). By investigating focusing efficiencies via ray simulations with optimized metalenses ((i) of Figure 4b), it turns out that refractive lens shows the best bandwidth performances. The comparison between diffractive a Fresnel lens and a metalens shown in sub-figure (ii) of Figure 4b reveals that a metalens exhibits wider angular bandwidth and narrow spectral bandwidth compared to diffractive lens.

**Figure 5:**
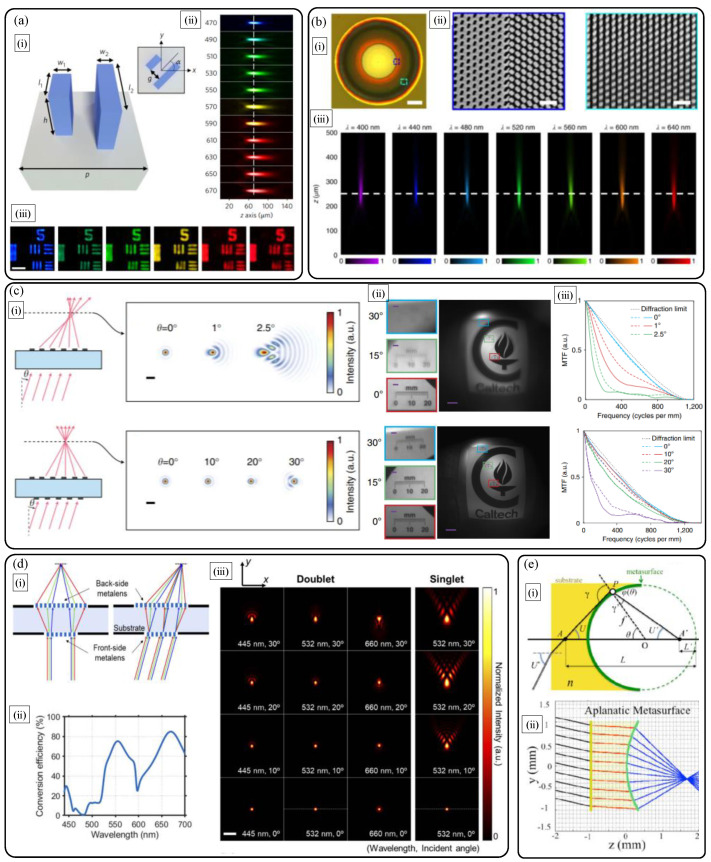
(**a**) Longitudinal chromatic aberration correction using anisotropic complex TiO_2_ nanofin library [19]. (**i**) Nanoantenna schematic, measured broadband achromatic (**ii**) focusing and (**iii**) imaging performances. (**b**) Longitudinal chromatic aberration correction with GaN metalens [20]. (**i**) Optical image and (**ii**) magnified scanning electron micrographs. (**iii**) Measured broadband achromatic focusing property. (**c**) Monolithic doublet metalens with a-Si nanoposts for reducing monochromatic aberrations [25]. Comparison of singlet and doublet in terms of (**i**) focusing and (**ii**), (**iii**) object imaging properties. (**ii**) Captured sample images and corresponding (**iii**) modulation transfer functions for sagittal (dashed lines) and tangential (solid lines) ray directions. (**d**) Doublet silicon metalens optimization for simultaneous correction of chromatic and monochromatic aberrations at the three colors [26]. (**i**) Concept diagram, (**ii**) color filtering efficiency of the backside metalens, and (**iii**) optimized performance comparison. (**e**) Curved aplanatic metalens design method [27]. (**i**) Schematic and (**ii**) deterministic design without spherical aberration and coma. Reprinted with permission from (**a**) [19] Copyright 2018 Springer Nature, (**b**) [20] Copyright 2018 Springer Nature, and (**c**) [25] Copyright 2016 Springer Nature. Reprinted with permission from (**d**) [26] Copyright The Optical Society and (**e**) [27] Copyright The Optical Society.

As discussed above, a dielectric metalens shows the severe chromatic and monochromatic aberrations. There have been many studies to compensate the aberrations. In particular, longitudinal chromatic aberration (LCA) of a dielectric metalens over the broad bandwidth has been thoroughly studied via polarization-dependent [19,20] (Figure 5a,b)) and -independent [21,22,23,24] phase encoding methods. The key points of such LCA compensation studies are the engineering of nanoantenna structures, simultaneously meeting required hyperbolic phase and spatial group delay map over a hyperbolic metalens at the design wavelength. To achieve such a goal, in 2018, W. T. Chen et al. investigated the library of complex TiO_2_ nanofin structures via numerous iterative electromagnetic simulations to secure enough nanoantenna building blocks, based on hybridization effect of the geometric phase and propagation phase (Figure 5a). However, owing to the simultaneous constraints of group delay and phase maps, the increase of NA and diameter are severely limited to compensate LCA over the broad visible bandwidth (full color). For 220 μm diameter, only the NA of 0.02 is demonstratred.

Through a similar approach, GaN nanopillar based broadband compensations of LCA were achieved by S. Wang et al. in 2018. Using GaN, focusing efficiency is a bit improved to 60% over the broad bandwidth (Figure 4b). However, fundamental limits in NA and diameter are also observed in this study and other polarization-independent metalenses [19,20,21,22,23,24]. These limits can be solved only by introducing new materials with higher refractive index and low extinction coefficients over the broad visible bandwidth, or delicate top-down fabrication of nanoantenna arrays with extremely large aspect ratio.

One of the major achievements in monochromatic aberration compensation in metalens is the doublet isotropic metalens [25]. Figure 5c describes that monochromatic aberration for large field angle, mainly coma, can be successfully compensated by patterning dielectric metalenses with optimized symmetric freeform phase profiles (not hyperbolic) at the two sides of a transparent substrate. By hybridizing the traditional doublet idea and combinatoric designs of the two phase maps (frontside and backside) and corresponding nanoantenna dimension, a wide angle ultracompact metalens camera is built for operation at a monochromatic light.

Inspired from the above early studies on chromatic and monochromatic aberration compensations, C. Kim et al. (Figure 5d) reported doublet metalens optimization research [26]. In the study, the authors found that simultaneous correction of LCA, transverse chromatic aberration (TCA), and the five monochromatic aberrations is almost impossible in the doublet p-Si metalens. The optimization goal of this study is set to correct LCA at the three colors of red, green, and blue, and monochromatic aberrations except distortion, simultaneously, by optimizing the two phase maps of metalenses. To achieve the physical encoding of such optimized phase maps, geometric-phase based backside metalens is optimized to show color filtering effect for noise cancellation. The frontside metalens is designed based on propagation phase encoding to correct aberration of the backside aberration. The results in Figure 5d shows that multiple aberrations are successfully corrected while retaining high NA (around 0.4) performance.

In addition, it is worth introducing the novel design idea using spherically curved metalens reported by Capasso’s group [27]. As depicted in Figure 5e, spherically curved metalens can efficiently correct spherical aberration and coma just by simple geometric optics calculation without iterative numerical computations. Via deterministic analytical calculation of optical phase difference condition using the generalized Snell’s law of refraction, aplanatic metalens phase map is simply calculated.

## 4. Applications Based on Three-Dimensional Imaging

In this section, recent studies on the three-dimensional imaging applications based on metalenses are introduced. The contents are classified into the two parts, display and sensing as follows.

### 4.1. Three-Dimensional Display

**Figure 6:**
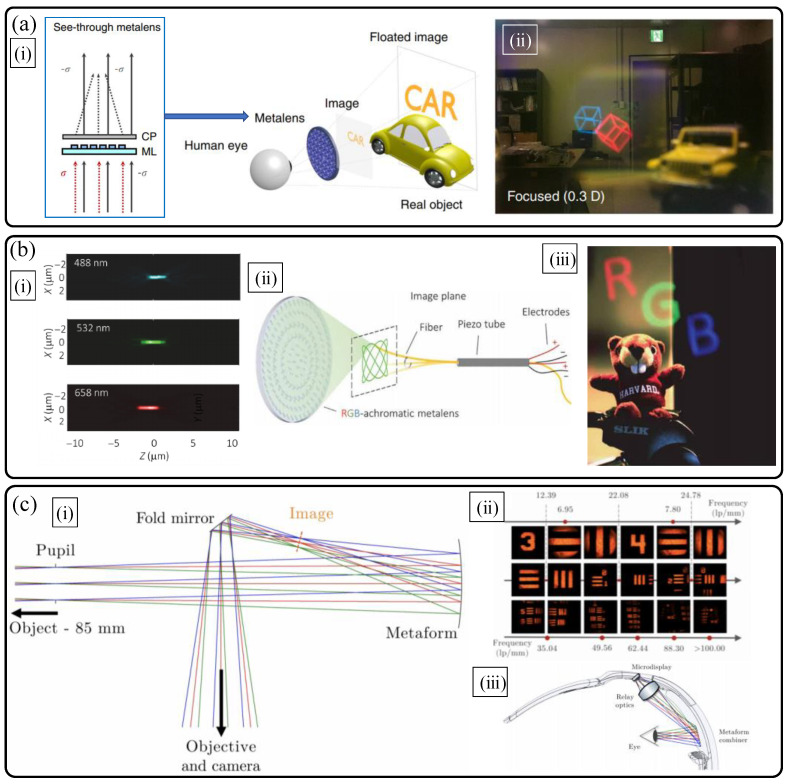
Metalenses for three-dimensional display applications: near-eye displays for augmented reality (AR) and virtual reality (VR). (**a**) Geometric phase based metasurface eyepiece as a see-through image combiner [28]. (**i**) Schematic diagram and (**ii**) captured full-color augmented reality scene with a camera. (**b**) Inverse designed metalens with chromatic aberration correction and its use in near-eye virtual image generation [29]. (**i**) Measured longitudinal apochromatic focusing property, (**ii**) principle of metalens assisted fiber scanning display, and (**iii**) captured image of full-color augmented reality scene using a beam splitter as an image combiner. (**c**) Hybridization of dielectric metasurface and freeform optics [30]. (**i**) Ray tracing diagram of a miniature image combiner design with a metaform mirror and (**ii**) the imaging result of a resolution target. (**iii**) A possible idea to use a metaform mirror as a see-through image combiner for a wearable near-eye display for AR curved eyeglass. (**a**) Reprinted with permission from [28] Copyright 2018 Springer Nature. Reprinted with permission from (**b**) from [29] and (**c**) from [30] Copyright 2021 The American Association for the Advancement of Science.

Among three-dimensional display applications, wearable near-eye displays for augmented reality (AR) and virtual reality (VR) with depth information have arisen as the most intriguing on-demand applications requiring extremely miniaturized advanced optical systems. In this context, there have been several high-quality studies of AR and VR displays using metalenses, and we would introduce the three different metasurface studies on the topic. Figure 6a describes an AR display system by G.-Y. Lee et al. [28] using a see-through metalens to combine a virtual images and background scenes. The use of transparent see-through dielectric geometric phase based metalens as an eyepiece in combination of a beam splitter (image combiner) allows an extremely large field of view (FOV) reaching 90 deg in the diagonal direction, and moderate transparency of background scene over broad bandwidth. The key features of an a-Si metalens eyepiece are judiciously engineered cross-polarization conversion efficiency for the formation of virtual images and transparency according to the handedness of circular polarization, and nanoimprint lithography-enabled large-scale fabrication (centimeter scale diameter). As a result, with high transparency, largely magnified virtual full-color three-dimensional videos are combined efficiently (Figure 6a).

Very recently, in 2021, Capasso’s group reported a flat optic AR/VR display system based on a novel full-color LCA compensated anisotropic metalens with high NA (0.7) and millimeter scale diameter [29]. It is worth noting that the newly suggested achromatic metalens design shows diffraction-limited high resolution focusing performances (the Strehl ratio over 0.97) for the full colors (red, green, and blue wavelengths). The novel inverse design idea of Fresnel-like multi-zone optimizations is suggested for full color LCA correction over a large diameter (possible even to centimeter scale). Figure 6b shows the correction results and its usage in AR/VR displays. As a display part, piezo-tube controlled compact fiber scanning display is adopted to generate full color virtual display. Then, the LCA corrected metalens could act as an eyepiece of full color AR and VR displays. However, owing to monochromatic off-axis aberrations and TCA, virtual images with large FOV were not demonstrated as the authors commented. In this work, quasi-depth cue generation is achieved with fast amplitude modulation of laser sources.

The last research to be introduced is about the hybridization of freeform optics and metasurface, so called meta-form optics, by D. K. Nikolov et al., published in 2021 [30]. The merits of freeform optics in advanced compact optical systems including AR and VR displays have been verified through many publications. Using freeform surfaces, many systems have shown diffraction-limited imaging performances in compact folded geometries [31,32]. Freeform optical surfaces are defined in complex manners without rotational invariance, and by virtue of the removal of symmetry in wavefront, complex ray path control and correction of multiple aberrations can be well achieved [33]. Thus, by placing a plasmonic metasurface on irregularly shaped curved freeform mirrors, a degree of freedom in design and modulation capability over a broad spatial frequency bandwidth could be achieved with extremely high design flexibility, light weight, and small form factor. In this meta-form optics paper, the combined benefits of the two technologies are verified with a design of extremely miniaturized folded imager and experimental demonstration for a monochromatic operation (Figure 6c).

In addition to the selected shape of freeform substrate (X-toroid) in the paper, there are many candidates of substrate geometry that can yield further design improvements. When it comes to the potential use of the metaform mirror for three-dimensional display, to demonstrate a monocular depth expression, continuous multi-focal imaging should be achieved. However, the loss of rotational symmetry of the freeform substrate hinders the high quality multi-focal design based on freeform optics, in general [31,32,33]. Hence, judicious design of metasurface should be integrated to a freeform substrate to improve such limitations in depth expression. Moreover, considering the sensitive alignment and resolution degradation issue followed, and metasurface demonstration methods (dielectric metasurface) suggested in the paper, it is expected that rigorous multi-scale studies in the topic would be required essentially for practical use of meta-form optics in advanced AR/VR three-dimensional displays.

### 4.2. Three-Dimensional Depth Sensing and Other Optical Sensing Applications

In this section, the recently reported advances in three-dimensional depth sensing and other optical sensing technologies using dielectric metalenses are introduced. First, we review the depth sensing methods using light field imaging principles [34,35], other methods based on point spread function (PSF) engineering [36,37], and cascaded metalens system based quantitative phase gradient microscopy [38].

The first category of metalens based depth imaging is based on spatial (array) and phase (interleaved) multiplexed metalens. Since the first proposals of light field camera by G. Lippmann in 1902 [39] and the plenoptic function by Adelson in 1991 [40], light field imaging technology based on ray optics received much attention in the field of modern optical engineering, for a simple and powerful method for extraction (sensing) and reconstruction (display) of three-dimensional object information. Light field cameras capture multiple elemental images of a certain object part, and such elemental images contain direction and intensity information in rays, rather than optical phase difference [41]. By capturing elemental image arrays, disparity and depth information can be extracted. The most general method to obtain elemental images is to use an imaging lens, a micro-lens array and a camera. However, intrinsically, using a micro-lens array (spatially multiplexed lenses) with a camera implies that resolution degradation is inevitable owing to low micro-lens NA and lowered effective pixel numbers in a camera.

**Figure 7:**
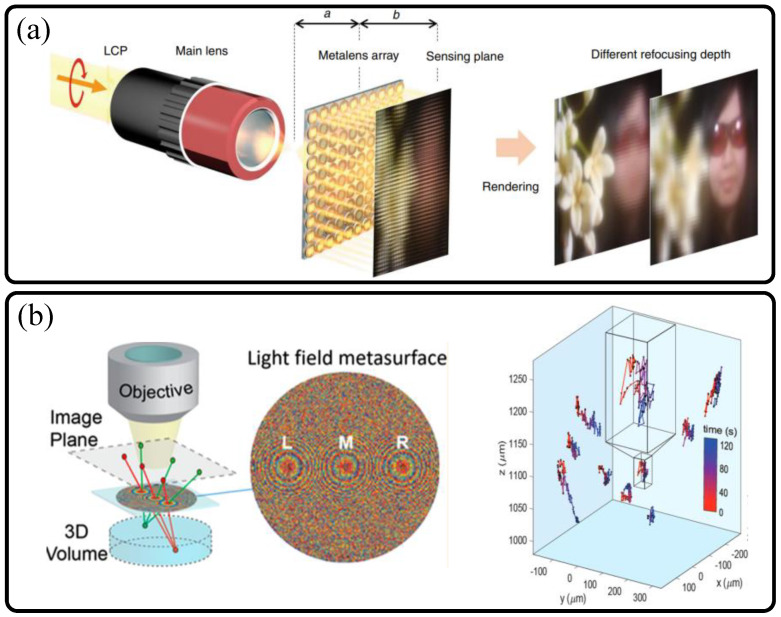
(**a**) Spatial [34] and (**b**) phase [35] multiplexed light field metasurfaces for 3D sensing applications. (**a**) Scheme of light field imaging for depth sensing using dielectric micro-metalens array. (**b**) Scheme of multi-foci light field silicon metalens for real-time 3D tracking of micro-beads with enhanced resolution (**left**), and experimental result (**right**). (**a**) Reprinted with permission from [34] Copyright 2019 Springer Nature. (**b**) Reprinted with permission from [35] Copyright 2019 American Chemical Society.

Here, we introduce two representative studies on metalenses for light field capturing with different design methods and goals. The first research depicted in Figure 7a was reported in 2019 by the Ding Ping Tsai group [34]. They utilized spatially multiplexed micro-metalens array with broadband LCA correction functions, which is discussed in the Section 3.2. Each elemental metalens of the array follows the Gaussian imaging relation as follows [34]:

(4) $$\frac{1}{a}+\frac{1}{b}=\frac{1}{f_{ml}}.$$

In Equation (4), *a*, *b*, and *f_ml_* refer to axial distance between an object and a metalens, axial distance between a metalens and an image, and focal length of a metalens, respectively. As LCA is the most critical aberration for low NA depth extraction via light field imaging with incoherent source, broadband achromatic metalens can play a vital role for this application with a small form factor. In a similar way with a conventional micro-refractive-lens array-based methods, FOV for depth extraction is retained. However, the inevitable degradation of imaging resolution is observed.

On the other hand, in 2019, the Brongersma group in Stanford University reported another approach of light field metasurface imaging for real-time 3D microscopy [35]. Their method exploits a tri-focal metalens with a same axial focal length via phase multiplexed metalens design by interleaving nanoantennas for the three unique phase maps of spherical focusing. As their goal was to track 3D positions of micron-scale florescent beads in real time beyond resolution trade-off (between axial and radial resolutions), the strategy of multi-functional shared-aperture metalens was highly effective (Figure 7b). The three different phase maps generate three different images of a single bead, and these images are captured by a single camera. Thus, in contrast to the research using a micro-metalens array, high spatial frequencies are conserved in imaging, and only expense is to sacrifice FOV. However, in this case, since imaging target is very small, reduction of FOV is not a critical issue.

**Figure 8:**
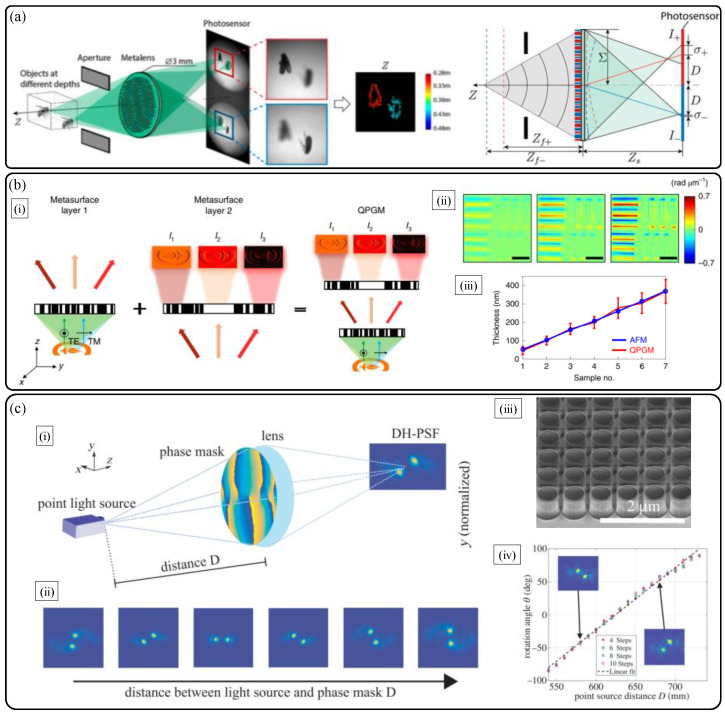
Other single-shot 3D sensing technologies with dielectric metalenses. (**a**) Single multiplexed metalens depth sensor inspired from eye structure of jumping spider [36]. (**b**) Monolithic doublet metalens based quantitative phase microscopy [38]. (**i**) Schematic diagram and (**ii**) phase retrieval result of a resolution target. (**iii**) Accurate depth sensing result in comparison with atomic force microscopy. (**c**) Phase mask optimized dielectric metalens depth sensor with engineered point spread function [37]. (**i**) Schematic diagram and (**ii**) working principle. (**iii**) Fabrication and (**iv**) depth sensing results. (**b**) Reprinted with permission from [38] Copyright 2019 Springer Nature. (**c**) Reprinted with permission from [37] Copyright 2019 SPIE.

There are several other noteworthy approaches for compact 3D depth sensing with metalenses based on a shared aperture metalens with engineered PSF [36], a complex metalens system based three-step phase shifting method [38], and a distance-dependently optimized PSF [37]. At first, we introduce a phase-multiplexed double foci TiO_2_ metalens with a shared aperture [36] reported in 2019. The key point of this method is to adopt an engineered double PSF metalens, whose images from a single point source are formed at the different spatial locations being separated along both transverse and longitudinal directions. Thus, two unique images with different defocus properties and perspectives are generated when a single 3D object is imaged via the metalens and a camera (Figure 8a). The method is similar to the aforementioned interleaved light field metalens based 3D particle tracking research. After capturing the dual images with different 2D intensity maps, a simple algorithm for aligning the images and extracting 2D depth map of the object is followed. The main advantage of the method is low computation load and a fast depth extraction speed with small form factor. The key idea of the algorithm is to conduct local differential computation about the relation between the designed PSFs’ widths and captured intensities as described in Equation (5) [36]:

(5) $$\frac{1}{\sigma }\frac{\partial I(x,y)}{\partial \sigma }=\nabla^{2}I(x,y).$$

In Equation (5), *I*(*x*,*y*) and *σ* are the captured object intensity and the PSF width at a camera plane, respectively. However, inherently, due to the analytical structure of the depth extracting algorithm from the two 2D images, it is hard to guarantee the prediction confidence for objects with a geometry of slowly varying depth map. For these structures, only the depth information of an object location, where intensity variation is not too small (around the object edges in general), is accurately predicted with depth resolution of about 5% of an object distance.

The second research by H. Kwon et al. [38] published in 2019 suggests a novel complex double-sided flat optic system with three optimized metalenses for single-shot phase imaging microscopy, which is briefly described in Figure 8b. The motif of this research originates from the well-known phase retrieval method of three-step phase shifting algorithm reported in 2006 [42]. The original motif work suggests obtaining a 2D phase map via three different interference intensity maps of a single object with different fringes rather than using time-consuming conventional unwrapping algorithms. However, to retrieve a phase map and follow depth information of certain transparent or oblique structures, three different phase intensity maps and bulky interferometer system are required as shown in following Equation (6) [42].

(6) $$\varphi (x,y)=\arctan\left(\sqrt{3}\frac{I_{1}-I_{3}}{2I_{2}-I_{1}-I_{3}}\right).$$

Here, *I*_2_ and *φ(x,y)* are the captured interference pattern of object and reference beam, and phase map of the object, respectively. *I*_1_ and *I*_3_ are the interference patterns captured when reference beam is shifted with lagging and leading phases with the same absolute value, respectively. In this study, quantitative phase gradient information is extracted based on such differential interference contrast method and three different interference patterns are generated by a double-sided monolithic tri-metalens system in a single shot ultracompact capture [38] without a bulky interferometry setup and a heavy computation load. The retrieved phase map can be utilized to extract height information of a sample with sub-micron scale axial resolution (Figure 8b).

There has been an intriguing study on double helix (DH)-PSF engineering of a dielectric metalens for depth map extraction by C. Jin et al., reported in 2019 [37]. Figure 8c illustrates that a metalens phase mask is optimized to demonstrate rotation of DH-PSF according to the change of axial distance between the object point and a metalens. A physical encoding of the metalens is conducted by using resonant Huygens’ cylindrical Si nanodisk arrays. Via the rotation angle of DH-PSH, distance information (centimeter scale) can be inferred. The drawback of this method is poor axial resolution and low NA (0.02) and FOV. The reason for the low NA is that the optimized DH-PSF operates only for highly paraxial diffraction waves due to paraxial resonance conditions of Huygens’ nanodisks.

**Figure 9:**
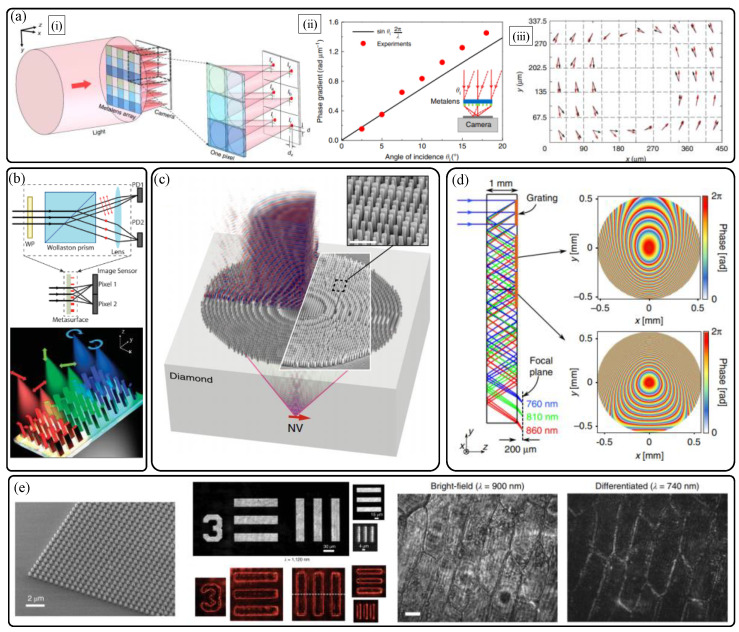
Various dielectric metalens sensing applications. (**a**) Micro-metalens array based polarimetric Shack-Hartmann wavefront sensor [43]. (**i**) Schematic diagram. Sensing results of (**ii**) angle of incidence and (**iii**) polarization map of azimuthally polarized Bessel beam. (**b**) Scheme of imaging polarimeter using micro-metalens array [44]. (**c**) Efficient quantum emitter sensing with immersion metalens [45]. (**d**) Folded meta-optics for compact spectrometer [46]. (**e**) Wavefront differentiation for edge detection using a Laplacian metasurface [47]. Fabrication result (**left**), edge detection of resolution target (**center**) and biological tissue (**right**). Reprinted with permission (**a**) from [43] Copyright 2018 Springer Nature, (**b**) from [44] Copyright 2018 American Chemical Society, (**c**) from [45] Copyright 2019 Springer Nature, (**d**) from [46] Copyright 2018 Springer Nature, and (**e**) from [47] Copyright 2020 Springer Nature.

The last part of the main section of this paper is a summarized introduction of other related optical sensing technologies. Figure 9 briefly introduce the five different studies of vectorial wavefront sensing [43], full Stokes parameter sensing of a 2D image [44], highly efficient quantum emitter sensing [45], ultracompact spectroscopy [46], and analog differentiation of optical wavefronts [47]. The conventional Shack–Hartmann wavefront sensing method is demonstrated with polarization-sensitive micro metalens arrays (Figure 9a) for sensing not only wavefront but also polarization map, simultaneously [43]. Single shot sensing method of a polarization map of a 2D image with full Stokes parameters including degree of polarization is also achieved by the Faraon group [44] (Figure 9b). Figure 9c illustrates the conceptual diagram of an immersion metalens based efficient sensing of quantum emitter (like nitrogen-vacancy center) in a high index substrate [45]. The high NA of such immersion metalens enables large outcoupling efficiency. It seems that such coupling between quantum emitters and an immersion metalens could be a new effective method to sense quantum states of emitters. By arranging multiple reflective metasurface gratings and lenses with a compact folded geometry, high resolution mobile ultracompact spectroscopy is also demonstrated (Figure 9d) [46]. By fine tuning of dispersions of multiple silicon metasurfaces, final focal points are judiciously designed to differ only in transverse direction in a focal plane with a detector, according to the input wavelength of illumination. The last research to be introduced is dielectric metasurface based analog wavefront processing [47]. The paper suggests that the Laplacian (2D second order differentiation) of the input wavefront is conducted optically without any digital processing, and edge detection of certain samples with high resolution is achieved by a simple combination of a conventional microscope and a designed metasurface (Figure 9e). There are also various interesting studies about metalens and metasurface applications which can extract various geometric information of objects [48,49,50,51,52,53,54,55,56,57] or reconstruct three-dimensional images for near-eye display [58,59,60], which are not discussed here.

## 5. Discussion

In this section, we suggest the two main challenges to improve performance and extend functionalities of the metalens, and related previous studies which have not been discussed above. One is to develop advanced numerical design methods of the metalens and metalens-based flat optic systems. Another is to develop active metalenses and metasurfaces with electrical tunability of encoded optical functions.

As introduced in Section 2, the basic design methods of dielectric metalens have been limited to the two semi-analytical approaches (geometric phase and propagation phase) in which dielectric nanoantennas are considered as single- or dual-mode waveguides. However, recently, the photonic inverse design methods by numerically optimizing arbitrary geometry of nanoantennas have been successfully verified via advanced electromagnetic optimization [61,62,63,64,65,66] and machine learning techniques [67,68,69]. Based on elaborate combination of the fast electromagnetic simulation methods and the robust frameworks of optimization and learning, improvements including bandwidth, NA, diffraction efficiency, and aberration correction have been achieved in a single flat optic device [61,62,63,64,65,66,67,68,69]. In the future, the authors expect that there will be many chances for the photonic inverse design methods to revolutionize three-dimensional display and sensing systems based on multiple flat optic elements.

While most studies on dielectric metalens and metasurface have been concentrated on passive dielectric metalenses whose optical functions are fixed once they are fabricated, active metasurfaces with reconfigurable functions still hold both tremendous potential and technical challenges [70]. In particular, metalenses with electrically tunable focal length and focusing efficiency have been in great demand in next-generation three-dimensional imaging applications such as multi-focal near eye displays, holographic displays, and compact depth sensing. Recently, there have been numerous studies on electrically tunable flat optics with advantages in large tunability and diffraction efficiency, such as thermo-optic polymer [71,72], micro-mechanical systems [73,74,75], deformable elastomer [76], twisted nematic liquid crystal [77,78,79], and phase-change materials [80,81,82,83]. For their practical use in ultracompact three-dimensional imaging applications, it seems that an active metalens that guarantees broadband visible range operation with high diffraction efficiency (transparency or reflectivity), and fast switching speed reaching ~kHz would be required. Moreover, to achieve such complex goals, large amounts of combinational studies involving novel material design, multi-physics device simulations, and delicate nanofabrication would be essential.

## 6. Conclusions

In this paper, dielectric metalens technology was thoroughly discussed in terms of the working principles, the basic performance characteristics of focusing and imaging, and the limits of performance originating from aberrations. Then, recent three-dimensional imaging applications based on dielectric metalenses were reviewed. The flat optics studies of three-dimensional display and sensing applications reveal that dielectric metalens technology hold promises of emerging tremendous opportunities for next-generation ultracompact optical systems beyond classical approaches. By deliberate hybridization with classical refractive and diffractive optic components, the authors envision that flat optic systems with dielectric metalenses can help in revolutionary miniaturization and multi-objective functional improvement of optical systems, simultaneously. Moreover, via inter-disciplinary research with the fields of computational imaging and vision, it is expected that a variety of advanced three-dimensional imaging applications would emerge with practical impact on our everyday life in the near future [84].
